# Bilateral Granulomatous Iridocyclitis Associated with Early-Onset Juvenile Psoriatic Arthritis

**DOI:** 10.1155/2022/3990406

**Published:** 2022-10-07

**Authors:** Christian Nieves-Ríos, Guillermo A. Requejo Figueroa, Sofía C. Ayala Rodríguez, Alejandra Santiago-Díaz, Eduardo J. Rodriguez-Garcia, Alejandro L. Perez, Erick Rivera-Grana, Adriana C. Figueroa-Díaz, Rafael Martín-García, Armando L. Oliver

**Affiliations:** ^1^Ponce Health Sciences University, Department of Surgery, Ponce, PR, USA; ^2^University of Puerto Rico School of Medicine, Department of Ophthalmology, Medical Sciences Campus, San Juan, PR, USA; ^3^University of Puerto Rico School of Medicine, Department of Dermatology, Medical Sciences Campus, San Juan, PR, USA

## Abstract

**Purpose:**

The purpose of this study is to report on a case of bilateral granulomatous iridocyclitis in a patient with early-onset juvenile psoriatic arthritis (JPsA).

**Methods:**

The method used is an observational case report. *Observations*. A 3-year-old Hispanic girl was sent to our uveitis service for further evaluation of her granulomatous uveitis. The initial ophthalmologic examination revealed bilateral band keratopathy, large mutton-fat keratic precipitates, multiple posterior synechiae, and 4+ anterior chamber cells. The physical exam was notable for left knee edema and right axillary rash. Laboratory testing was remarkable for an erythrocyte sedimentation rate of 80 mm/h, positive antinuclear antibodies (1 : 1, 280), and negative human leukocyte antigen B27. A cutaneous biopsy was obtained, which confirmed the diagnosis of a psoriatic rash. Treatment with oral prednisolone and topical prednisolone acetate with atropine sulfate resulted in the complete resolution of the uveitis. *Conclusion and Importance*. Bilateral granulomatous iridocyclitis may be a rare presentation of ocular involvement in patients with early-onset JPsA.

## 1. Introduction

Uveitis has been documented as one of the most common extra-articular manifestations in patients with psoriatic arthritis (PsA) [1–4]. In the pediatric population, it has been shown that juvenile PsA (JPsA) is more frequently associated with uveitis than other subgroups of juvenile idiopathic arthritis (JIA) [5], with an estimated prevalence between 7 to 21% [3, 5–8]. Moreover, the characterization of uveitis in children with PsA indicates that it has noticeable distinctions from the uveitis seen in adults with PsA [9].

JPsA can be divided into 2 clinical subgroups that are based on age at disease onset [3, 6, 9]. Patients with early-onset JPsA are more likely to be female and express antinuclear antibodies (ANAs) [6, 10], and they are at a higher risk for ocular involvement [3, 10]. Uveitis in this subgroup is currently characterized as bilateral, chronic, anterior, and/or intermediate [2, 9]. However, a detailed description of uveitis in JPsA is limited due to its rarity [2].

Most of the previously reported cases of JPsA-associated uveitis have been clinically described as nongranulomatous [2, 3, 7–9, 11–18]. However, for JIA-associated uveitis in general, recent studies have shown that granulomatous uveitis may be more common than previously thought [19, 20]. Along those lines, we present a rare case of bilateral granulomatous iridocyclitis associated with early-onset JPsA.

## 2. Case Presentation

A 3-year-old Hispanic girl with no prior history of systemic disease was sent for a consultation to our uveitis service for the further evaluation of her bilateral granulomatous uveitis. Her family history was remarkable for PsA in her father and uncle. The review of systems revealed that she had a 3-month history of a skin eruption and left knee arthralgia.

**Figure 1:**
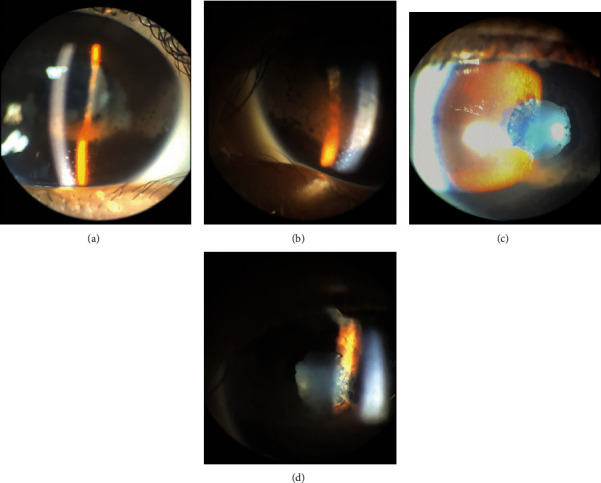
Color photographs (obtained upon presentation) showing granulomatous (mutton-fat) KPs, band keratopathies (a and b), and multiple posterior synechiae (c and d); OD and OS, respectively.

Upon a comprehensive examination, her best-corrected visual acuity (BCVA) was 20/60 in both eyes. The intraocular pressure was 13 mmHg in the right eye (OD) and 14 mmHg in the left eye (OS). A slit-lamp exam revealed bilateral band keratopathy, large mutton-fat keratic precipitates (KPs), multiple posterior synechiae (Figures 1(a)–1(d)), and 4+ anterior chamber cells. The fundus view was very poor for both eyes due to the presence of band keratopathy and limited pupillary dilation. A B-scan ultrasound was unremarkable, with no evidence of vitreous opacity.

**Figure 2:**
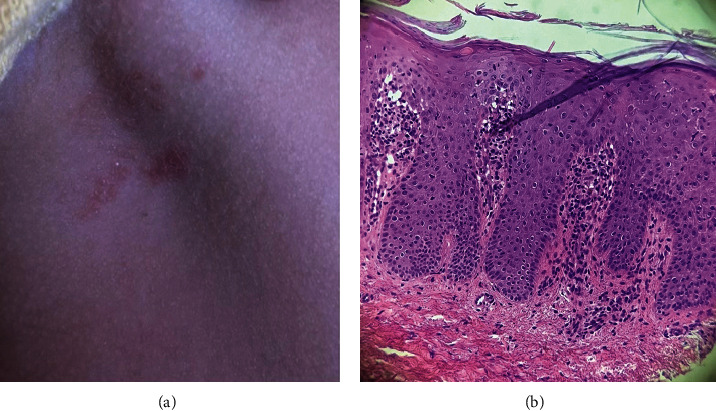
Erythematous papules and plaques with thin overlying scales on the right axilla (a). Histopathological examination (H&E stain, 200X) demonstrates psoriasiform acanthosis, thin supra-papillary plates, neutrophils in the stratum corneum, and a superficial perivascular lymphocytic infiltrate, confirming the diagnosis of psoriasis (b). H&E: hematoxylin and eosin.

The physical examination was remarkable for left knee edema and a right axillary nonpruritic eruption consisting of irregularly shaped erythematous papules and plaques with thin scale (Figure 2(a)). No dactylitis, enthesitis, or nail changes were identified. As the differential diagnosis of granulomatous iridocyclitis includes infectious and noninfectious etiologies, a diagnostic work-up was ordered, and the patient was started on hourly topical prednisolone acetate 1% and atropine sulfate 1%, the latter, twice a day.

The radiological images were remarkable only for left knee effusion. The girl's chest X-ray was normal, with no lymphadenopathy, opacities, or cavitations. Routine laboratory tests revealed an erythrocyte sedimentation rate of 80 mm/h and an unremarkable complete metabolic panel (CMP) (Na: 141 mEq/L, K: 3.7 mEq/L, Cl: 103 mEq/L, blood urea nitrogen: 9.00 mg/dL, and serum creatinine: 0.25 mg/dL) and urinalysis (negative for glucose, protein, red blood cells, white blood cells, and casts). Her immunology work-up was remarkable for a positive ANA test (1 : 1,280), with a homogenous pattern and negative anti-double-stranded DNA antibody, anti-Smith antibody, rheumatoid factor (<10 IU/mL), and cyclic citrullinated peptide IgG. Fluorescent treponemal antibody absorption (FTA-ABS), venereal disease research laboratory (VDRL), and tuberculosis interferon-gamma release assay (IGRA) tests were negative. Serological tests for the human leukocyte antigen B27 haplotype, HIV, hepatitis A IgM, hepatitis B core IgG, and the hepatitis C antibody were negative.

A punch biopsy of a skin lesion revealed psoriasiform acanthosis, thin supra-papillary plates, neutrophils in the stratum corneum, and superficial perivascular lymphocytic infiltrate (Figure 2(b)). Based on these results, the uveitis specialist concluded a diagnosis of bilateral granulomatous iridocyclitis associated with early-onset JPsA.

After establishing a tissue diagnosis of JPsA, oral prednisolone (1 mg/kg) was added to her therapy. Following 2 weeks of treatment, her uveitis was inactive, bilaterally. The BCVA improved to 20/40 in OD and remained stable at 20/60 in OS. At the 2-month follow-up visit, her uveitis remained inactive, and her BCVA improved to 20/30 in OD and 20/40 in OS. At this visit, a standardized prednisolone tapering protocol was begun, and she was started on subcutaneous methotrexate (0.2 mg/kg/week) as a corticosteroid-sparing therapy.

## 3. Discussion

To the best of our knowledge, as of the writing of this manuscript, this case represents the first report of early-onset JPsA associated with bilateral granulomatous iridocyclitis. In addition, our findings are in agreement with those of an increasing number of reports revealing the presence of granulomatous manifestations in JIA-related uveitis (with reported prevalences ranging from 7 to 32%) [19–21].

Uveitis in adults with PsA has been studied extensively; however, much less is known about its presentation in JPsA [3, 9]. A case series from Salek et al. was the first article to characterize uveitis in childhood PsA [9]. Although the sample size was small, Salek and his team described uveitis in children with early-onset (6 years old or younger) JPsA as being bilateral, chronic, anterior, and/or intermediate [9]. In their study, 5 of the 10 affected eyes developed band keratopathy [9], a complication typically associated with chronic intraocular inflammation [22]. In a recent study by Baquet-Walscheid et al. on JPsA, most of the participating patients presented with insidious, anterior uveitis that could be either unilateral or bilateral [3]. However, ophthalmological descriptions were available for only 18% of the patients with JPsA-related uveitis and were not stratified by the age of onset. Moreover, none of these studies specified whether the ocular findings were consistent with granulomatous or nongranulomatous uveitis.

Juvenile psoriatic arthritis exhibits a biphasic age of onset, in which distinguishing clinical differences can be identified between both age groups [3, 6, 10]. Previous studies noted that patients with early-onset JPsA were more often female and ANA positive [6], had a higher risk for uveitis, and were less frequently diagnosed with psoriasis [3, 10]. With its relatively smaller plaques and thinner scales, childhood psoriasis may present more subtly than does adult plaque psoriasis. Involvement of the scalp, face, and flexural areas is also more characteristic of childhood-onset psoriasis [17, 23].

According to a study that examined systemic manifestations of sarcoidosis in patients with ocular sarcoid uveitis, cutaneous sarcoid was the second most common systemic manifestation [24]. In that line, we considered obtaining a punch biopsy of the patient's rash to rule out conditions including sarcoidosis (which can be associated with granulomatous uveitis) [24, 25]. As the histological analysis results were consistent with psoriasis, we concluded a diagnosis of granulomatous iridocyclitis associated with JPsA. On the other hand, given the clinical presentation, the unremarkable chest X-ray (without bilateral adenopathy or opacities), and the patient's young age (3-year-old), we did not order a chest CT scan to limit the patient's radiation exposure. Moreover, previous studies have shown that sarcoid arthritis is an uncommon manifestation of systemic sarcoidosis, typically reported in 5% or less [24, 26], making it less likely to be the cause in our case.

On the other hand, a urinalysis and CMP were ordered to evaluate renal function and rule out tubulointerstitial nephritis and uveitis (TINU) syndrome. Patients with TINU can present at a young age with bilateral anterior uveitis [27, 28]. However, uveitis in TINU has been mainly characterized as nongranulomatous and associated with an acute presentation of eye redness, pain, and photophobia [27, 28].

Salek et al. also noted that children with early-onset JPsA can develop a severe form of uveitis that may require management with multiple immunosuppressive medications [9]. In their study, they found that all the patients required the addition of a biologic agent, even after having received treatment with methotrexate and some form of corticosteroid [9]. Similarly, Moretti et al. described a case of early-onset JPsA associated with refractory uveitis [14]. In this case, the child failed to respond to nonsteroidal anti-inflammatory drugs, methotrexate, and etanercept, ultimately requiring the use of adalimumab to achieve sustained clinical remission [14]. These findings are in concordance with those of the SYCAMORE trial on JIA-related uveitis, in which management with adalimumab plus methotrexate was associated with a lower rate of treatment failure compared to management with methotrexate alone [29]. Hence, biological therapies may serve as valuable alternatives when there is an insufficient response to conventional treatment [30]. In conclusion, bilateral granulomatous iridocyclitis may be a rare presentation of ocular involvement in patients with early-onset JPsA. However, young patients with bilateral uveitis should be evaluated for other possible systemic associations, such as sarcoidosis and TINU syndrome. As highlighted in our case, a cutaneous biopsy may help establish a tissue diagnosis for patients with uveitis, particularly those with skin manifestations [31, 32].

## Data Availability

There is no data set to be submitted with this manuscript.
